# Cytological Features of Mammary Analogue Secretory Carcinoma of the Parotid Gland in a 15-Year-Old Girl: A Case Report with Review of the Literature

**DOI:** 10.1155/2015/656107

**Published:** 2015-03-01

**Authors:** Takako Inaba, Yuki Fukumura, Tsuyoshi Saito, Junkichi Yokoyama, Shinichi Ohba, Atsushi Arakawa, Takashi Yao

**Affiliations:** ^1^Division of Diagnostic Pathology, Juntendo University, 3-1-3 Hongo, Bunkyo-ku, Tokyo 113-0033, Japan; ^2^Department of Human Pathology, Juntendo University School of Medicine, 1-1-19 Motomachi Bldg. 3F, Hongo, Bunkyo-ku, Tokyo 113-0033, Japan; ^3^Department of Otorhinolaryngology, Juntendo University School of Medicine, 3-1-3 Hongo, Bunkyo-ku, Tokyo 113-0033, Japan

## Abstract

Mammary analogue secretory carcinoma (MASC) is a recently recognized tumor of salivary glands characterized by the ETV6-NTRK3 fusion gene. This tumor is very rare in children and adolescents. We report a case of MASC in a 15-year-old girl, the fifth youngest case so far reported. The patient complained of a left infra-auricular mass that gradually enlarged for a year. Fine-needle aspiration cytology/imprint cytology showed individual tumor cells that had faintly eosinophilic granular cytoplasm with secretion granules sometimes seen adjacent to the tumor cells. These cytological features overlapped between those of zymogen granule-poor acinic cell carcinoma (AciCC) and MASC. In addition to the case report, we present a review of the related literature with a focus on the cytological features of MASC. The differential diagnostic clues are also discussed.

## 1. Introduction

Malignant tumors of the salivary gland are rare in the pediatric population. Of these, the most common histological types are mucoepidermoid carcinoma (MEC) and acinic cell carcinoma (AciCC) [1]. Mammary analogue secretory carcinoma (MASC) is a recently established salivary gland tumor, and it has been shown to affect both adult and child populations [2].

Herein, we report a case of parotid gland MASC in a 15-year-old girl along with a review of the literature on MASC in the young. Our case was cytologically diagnosed as “ACiCC, mostly composed of intercalated duct cell type.” After surgical resection, it was successfully diagnosed as MASC of the parotid gland, by the detection of an* ETV6-NTRK3* fusion gene by RT-PCR. A retrospective review of the cytology indicated convincing features of MASC. We discuss here what could aid the diagnosis of MASC and the differentiation from acinic cell carcinoma (ACiCC), one of the most difficult salivary gland tumors to differentiate. As far as we know, only 7 cases of MASC have been reported with molecular confirmation below 19 years of age [3–8], and this is the 5th youngest case of it.

## 2. Case Presentation

**Figure 1:**
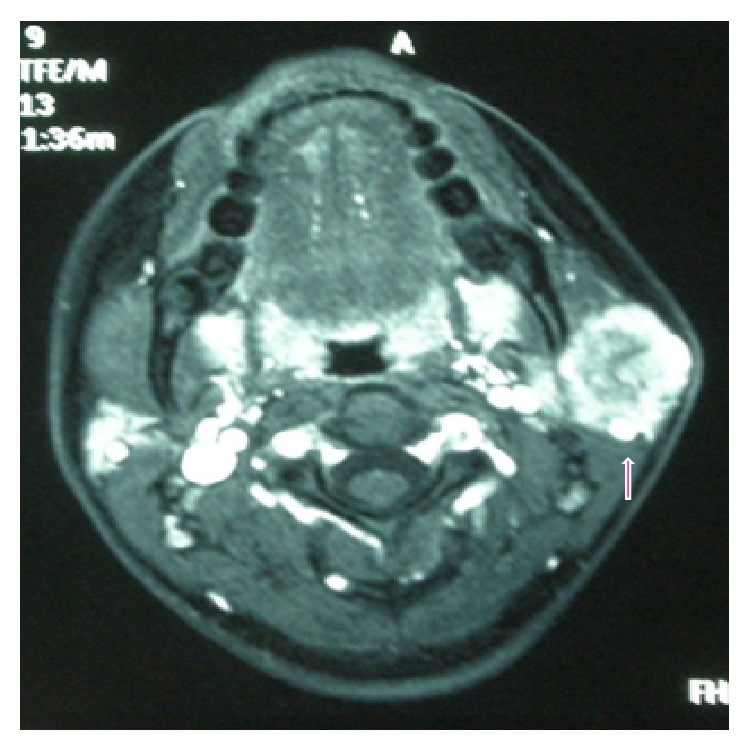
Contrast-enhanced computed tomography images of the tumor. A 2.8 cm sized tumor was present at the left parotid gland. The tumor was relatively circumscribed and contained a low-density area inside.

A 15-year-old girl visited hospital for an otorhinolaryngology consultation because of a left infra-auricular mass, which had shown gradual enlargement for a year. Ultrasound-guided fine-needle aspiration (FNA) was performed and the diagnosis of possible ACiCC was rendered. The patient was introduced to our hospital in order to proceed onto surgery. The FNA materials obtained at the former hospital, consisting of 2 Papanicolaou-stained slides and 1 Giemsa-stained slide, were submitted to our hospital and ACiCC was again most strongly suspected by our cytological diagnosis. A CT scan showed a 2.8 cm sized mass in the left parotid gland (Figure 1). The patient underwent excision of her whole left parotid gland and imprint cytology was also obtained intraoperatively. This case had no metastasis in the regional lymph nodes of levels 1–4 and was staged as T1N0M0, Stage I. No adjuvant therapy was given, and the patient has no evidence of disease 40 months after surgery.

### 2.1. FNA and Imprint Cytology

**Figure 2:**
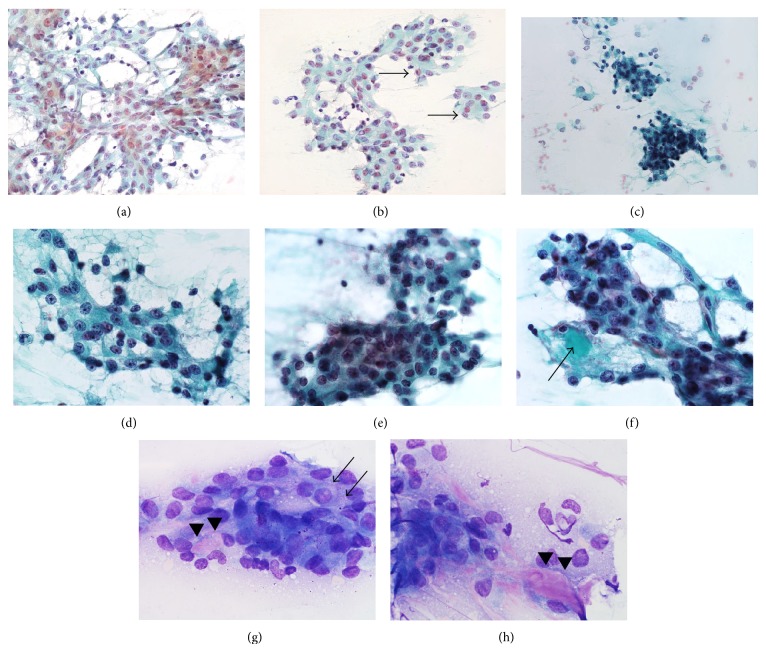
Cytomorphology of mammary analogue secretory carcinoma. Tumor cells were seen in a relatively flat sheet with abundant vascular supply (a). Tubules or rosette-like structures were sometimes seen (arrows, (b)). Tumor cells sometimes piled up with scattered detached cells peripherally (c). The cytoplasm of the tumor cells was slightly eosinophilic and the cell border between the tumor cells was indistinct ((d), (e)). Light-greenish material was sometimes observed at the periphery of the tumor cell cluster (arrow), which is now considered to correspond to material secreted by the tumor cells (f). With Giemsa stain, tumor cells showed slightly eosinophilic cytoplasm and intracytoplasmic granules (arrows) were rarely seen (g). Eosinophilic substance (arrowheads) showing slight metachromacy was seen inside tumor tubules/in the background ((g), (h)). (a) to (f) Papanicolaou stain; (a) and (b) FNA material; (c) to (f) imprint material; (g) and (h) Giemsa stain.

The FNA specimen and imprint specimen showed similar cytological features; that is, the architecture of cells, individual cell appearance, and background were very similar between them. The cell clusters were mostly thin sheet-like, sometimes with slight accumulation, or were seen in an arborizing pattern with a vascular core (Figure 2(a)). Tubule formation was sometimes seen (Figure 2(b)). The cell adhesion between the tumor cells looked rather loose and they seemed easy to detach (Figure 2(c)). The cell border between the tumor cells was indistinct (Figure 2(d)). Individual tumor cells had slightly eosinophilic and granular cytoplasm (Figures 2(d) and 2(e)). Cytoplasmic vacuoles were not remarkable. Tumor cells were relatively uniform throughout, with little cellular pleomorphism. The nuclei of the tumor cells were round with no distortion and located mostly peripherally, containing small nucleoli. Although it was not recognized as such at the time of cytological diagnosis, retrospective review sometimes revealed a light-greenish material corresponding to material secreted by the tumor cells attached to the cell cluster (Figure 2(f)). Giemsa-stained specimen also showed that tumor cells had slightly eosinophilic and granular cytoplasm (Figure 2(g)). Pinkish material showing slight metachromacy was seen inside tubular structures/in the background (Figures 2(f) and 2(g)).

### 2.2. Surgical Specimen

**Figure 3:**
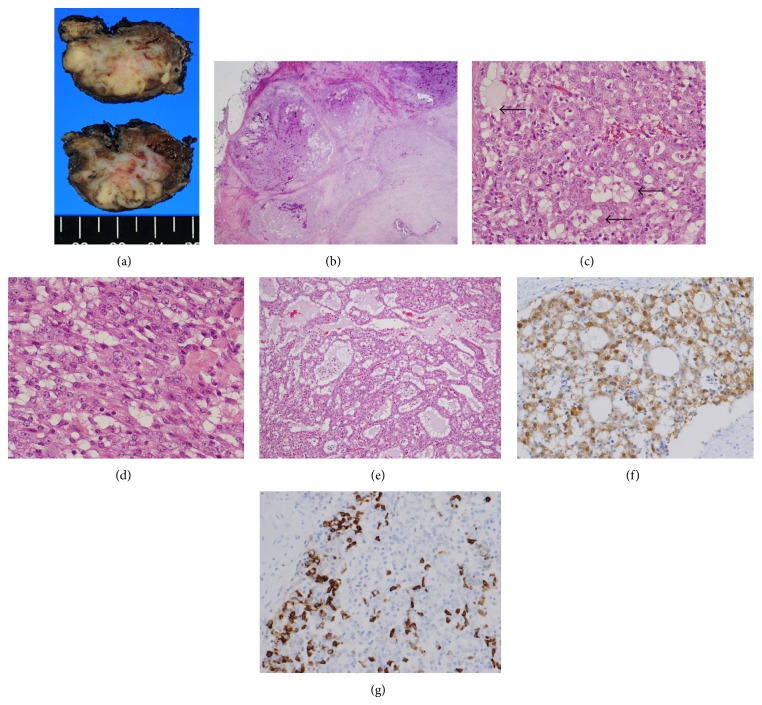
Macroscopic and histological morphology of mammary analogue secretory carcinoma. The cut section of the tumor was uncircumscribed and whitish, invading into the connective tissue surrounding the parotid gland (a). Microscopically, the tumor grew in a nodular fashion with abundant fibrous septa (b). The tumor formed small ducts or microcysts and often had vacuolated cytoplasm (arrows) (c). Tumor cells were relatively uniform and showed morphology similar to intercalated duct (d). Pinkish/mucin-like substances were sometimes seen inside the tumor tubules (e). Immunohistochemically, tumor cells were diffusely and intensely positive for S-100 (f) and partially/intensely positive for mammaglobin (g). (b) to (e) HE stain; (f) immunohistochemistry for S100; (g) immunohistochemistry for mammaglobin.

Cut sections of the surgical specimen showed a 3.0 × 2.8 × 2.5-cm sized, uncircumscribed, whitish, elastic hard mass in both lobes of the left parotid gland (Figure 3(a)). Histopathologically, the tumor grew partially with a multinodular appearance but often showed invasive growth with fibrotic/hyalinized stroma (Figure 3(b)). In the tumor nodule, tumor cells formed small tubules or acini sometimes with secretory material inside (Figure 3(c)). Tumor cells were relatively uniform, similar to intercalated duct cells, and often had vacuolated cytoplasm (Figure 3(d)). A small portion of the tumor showed the aggregates of irregularly dilated glands with secretory material inside (Figure 3(e)). Only a few mitotic figures were observed. No lymphatic/vascular or perineural invasion was evident.

Periodic acid Schiff (PAS) stain showed positivity for the eosinophilic material inside the dilated tubule and the luminal site of the tumor tubules, but negativity for the cytoplasm, suggesting a lack of zymogen granules. Immunohistochemically, tumor cells were intensely positive for S-100 (Figure 3(f)), GCDFP-15, and mammaglobin (Figure 3(g)), while ER, PgR, HER2, and p63 were negative. The Ki-67 index of the tumor was 22%.

### 2.3. Molecular Pathology

**Figure 4:**
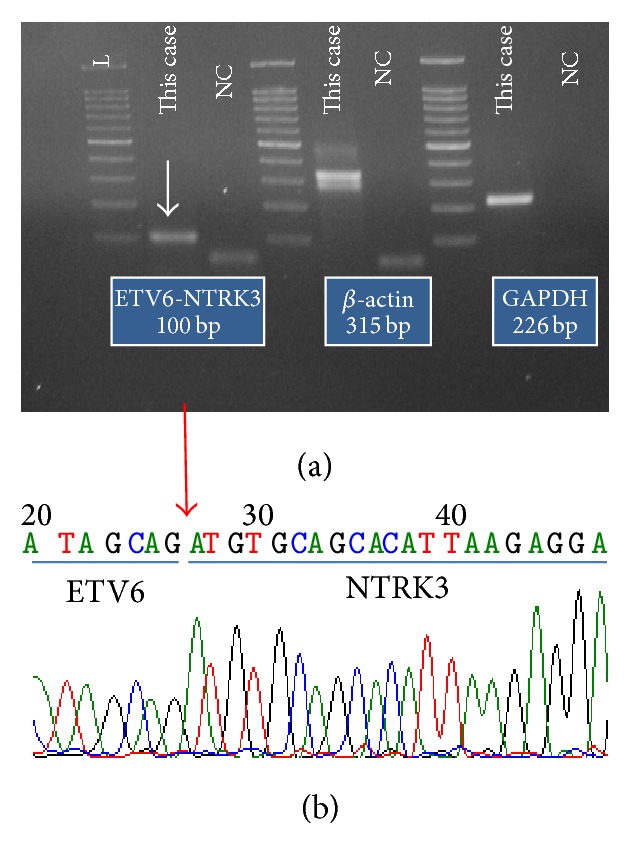
The analysis of* ETV6-NTRK3* fusion transcript. The results of the electrophoresis (a) and direct sequencing (b) revealed the presence of an* ETV6-NTRK3* fusion gene. In (a), the arrow indicates the PCR product of the* ETV6-NTRK3* transcript. PCR for *β*-*actin* and* GAPDH* was performed as a quality check of the extracted RNA. NC: negative control. L: ladder (molecular marker). In (b), the arrow indicates the fusion point of the* ETV6-NTRK3* transcript.

A formalin-fixed, paraffin-embedded section obtained from the surgical specimen was submitted for RT-PCR studies. RNA was extracted and converted into cDNA. RT-PCR was performed using previously described primer pairs [9]. This analysis revealed the presence of an* ETV6-NTRK3* fusion transcript in this tumor (Figures 4(a) and 4(b)). The final diagnosis was MASC.

## 3. Discussion

MASC was first described by Skálová et al. in 2010 [9] and more than 90 cases have been reported in the last 2 years [2]. Unlike secretory breast carcinoma, which is usually seen in young patients, MASC occurs in both children and adults (13 to 72 years), with an average age of 44.2 years, and shows slight male predominance. The present case was a 15-year-old female with the complaint of an infra-auricular mass/swelling for one year and is the fifth youngest case of MASC so far reported with molecular conformation (Table 1). Although relatively rare, we need to keep this entity in mind for the differential diagnosis of salivary gland tumors in pediatric patients. In pediatrics, the majority of MASC occur in the parotid gland, but they can occur in other locations, such as lips, and are most often diagnosed as AciCC before molecular study [3–8].

The cytological distinction between zymogen granule-poor ACiCC and MASC without molecular study or immunohistochemical study is very difficult, since the cytology of MASC has many overlapping features with AciCC. However, there seem to be several cytological features suggestive of MASC, such as papillary structure, extracellular and intracellular mucinous material, vacuolated cytoplasm, tumor cells with low-grade nuclear atypia, and abundant vascularity [3–8, 10–12]. Retrospectively, three out of five of the above-mentioned features, mucinous material (extracellular, in this case), low-grade nuclear atypia, and abundant vascularity, were seen in the present case, which might have led to the diagnosis of MASC. According to Griffith et al., the subtle clues for discriminating MASC from ACiCC are prominent extracellular and intracellular mucin, greater size variability of cytoplasmic vacuoles, and clinical information such as the tumor's anatomic site and the sex of the patient [7]. The absence of basophilic cytoplasmic granules seen in ACiCC is also important.

In MASC, it is described that perineural invasion is uncommon and that lymphovascular invasion has not been reported; therefore, MASC is currently regarded as a low-grade carcinoma, and its prognosis appears to be favorable [9]. The present case is alive and well with no evidence of disease 40 months after surgery. Skálová et al. recently showed that MASC can transform into a high-grade tumor [13]. Therefore, it should be considered by cytologists that tumor cells with high-grade nuclear atypia might appear in the cytological specimen of MASC.

In conclusion, the cytological features of MASC of the salivary gland of a 15-year-old girl were reported. Since preoperative FNA is increasingly and widely used for tumors of the salivary gland owing to its simplicity and safety, it is necessary for cytologists and pathologists to be aware of the cytology of recently established tumors such as MASC.

To the best of our knowledge, this is the fifth youngest case of MASC reported in the literature.

## Figures and Tables

**Table 1:** Mammary analogue secretory carcinoma cases under 19 years old: summary of the reported cases (with molecular confirmation).

Age	Sex	Site^a^	Size^b^	Duration^c^	Outcome/FU^d^	Original Dx./procedure^e^	Others^f^	Reference
13	M	P	2.1	3 yr	No rec/8 mo	AciCC/His		[3]
14	M	P	2.0	1 yr	NA	Sal Neopl/His	Post-rad	[4]
14	F	P	2.8	NA	NA	AciCC/His		[5]
14	F	P	3.0	4 mo	No rec/1 yr	AciCC/His		[6]
15	F	P	3.0	1 yr	No rec	AciCC/FNA & His		This case
15	M	Lip, sp.	1.0	NA	NA	Neopl.NOS/NA		[7]
17	F	P	1.0	NA	Rec^g^	MEC/FNA		[8]
17	M	P	1.5	NA	No rec/NA	Pleo/FNA		[8]

^a^P: parotid gland; Lip, sp.: upper lip.

^b^Size in cm.

^c^Duration: duration before surgery; NA: duration before surgery was not available.

^d^rec: recurrence; No rec/NA: no recurrence/follow-up period is not available.

^e^AciCC: acinic cell carcinoma; Sal Neopl: salivary neoplasm with no further classification; Neopl NOS: neoplasm NOS; MEC: mucoepidermoid carcinoma; Pleo: pleomorphic adenoma; NA: not written in the literature; for procedure; His: by histology; FNA: by fine-needle aspiration cytology.

^f^Post-rad: postradiation therapy.

^g^Recurred 6 times during the 15 years after first surgery according to [8].
